# The Trends in Cardiovascular Diseases and Respiratory Diseases Mortality in Urban and Rural China, 1990–2015

**DOI:** 10.3390/ijerph14111391

**Published:** 2017-11-15

**Authors:** Weiwei Sun, Yun Zhou, Zhuang Zhang, Limin Cao, Weihong Chen

**Affiliations:** 1 Department of Occupational & Environmental Health, School of Public Health, Tongji Medical College, Huazhong University of Science and Technology, Wuhan 430030, China; m201575184@hust.edu.cn (W.S.); 2017220153@hust.edu.cn (Y.Z.); d201781173@hust.edu.cn (Z.Z.); d201578078@hust.edu.cn (L.C.); 2 Key Laboratory of Environment and Health, Ministry of Education & Ministry of Environmental Protection, and State Key Laboratory of Environmental Health (Incubating), School of Public Health, Tongji Medical College, Huazhong University of Science and Technology, Wuhan 430030, China

**Keywords:** cardiovascular diseases, respiratory diseases, mortality, years of life lost, burden of disease, trends

## Abstract

With the rapid development of the economy over the past 20 years, the mortality rates from cardiovascular diseases (CVDs) and respiratory diseases (RDs) have changed in China. This study aimed to analyze the trends of mortality rates and years of life lost (YLLs) from CVDs and RDs in the rural and urban population from 1990 to 2015. Using data from Chinese yearbooks, joinpoint regression analysis was employed to estimate the annual percent change (APC) of mortality rates from CVDs and RDs. YLLs due to CVDs and RDs were calculated by a standard method, adopting recommended standard life expectancy at birth values of 80 years for men and 82.5 years for women. Age-standardized mortality rates and YLL rates were calculated by using the direct method based on the Chinese population from the sixth population census of 2010. Age-standardized mortality rates from CVDs for urban residents and from RDs for both urban and rural residents showed decreasing trends in China from 1990 to 2015. Age-standardized mortality rates from CVDs among rural residents remained constant during above period and outstripped those among urban residents gradually. The age-standardized YLL rates of CVDs for urban and rural residents decreased 35.2% and 8.3% respectively. Additionally, the age-standardized YLL rates of RDs for urban and rural residents decreased 64.2% and 79.0% respectively. The age-standardized mortality and YLL rates from CVDs and RDs gradually decreased in China from 1990 to 2015. We observed more substantial declines of the mortality rates from CVDs in urban areas and from RDs in rural areas.

## 1. Introduction

Cardiovascular diseases (CVDs) and respiratory diseases (RDs) are two of the most common diseases in the world [1]. The Global Burden of Disease Study indicated that CVDs caused 17.9 million deaths, and chronic respiratory diseases caused 3.8 million deaths in 2015 worldwide [2]. CVDs were the most common cause of death in Chinese population in 2015, followed by cancer and RDs [3]. In recent years, these two disease categories were responsible for more than 50% of deaths in China (CVDs 45% and RDs 11% in 2014) and more than double the proportion of deaths from neoplasms (23%) [4]. Due to huge health burden induced by these two kinds of diseases, they are public health problem and the major prevention and control program targets in China. Therefore, further analysis of the trends on the mortality rates from CVDs and RDs is essential for disease control.

The age-standardized death rates from CVDs and RDs have declined dramatically worldwide in the past 20 years [5], while the trend and magnitude has varied in different countries [6,7]. China has experienced rapid economic development, including industrialization and transportation development during this period. With the economic development, the medical service system has been greatly improved, especially in rural areas. The overall health condition in China has improved, the mortality from infectious diseases is declining rapidly, and the life expectancy has increased 9.5 years from 1990 to 2015 [2].

However, as food availability has expanded and become more diversified, it has adversely affected health on account of inappropriate dietary patterns. Unhealthy diets, such as ones high in fat and sodium, and low in fruit and vegetables, are considered to be the main cause of CVDs [8,9]. Furthermore, environmental risk factors also play a dominant role in the disease development. The World Health Organization has estimated that air pollution was associated with 1.6 million premature deaths per year in China [10]. The air pollution is becoming more and more serious in many cities and the areas surrounding industrial enterprises. In addition, accumulating evidence suggests that exposure to air pollutants is associated with elevated mortality and morbidity from CVDs and RDs [11,12]. The influence of lifestyle, environmental pollution and medical service maybe appear through the trends of the mortality rates during last 20 years. Years of life lost (YLLs) are used here to capture premature mortality due to CVDs and RDs.

This study aimed to provide a comprehensive profile of the mortality trends and YLLs from CVDs and RDs in China from 1990 to 2015. We also compared the disparity of mortality rates and YLLs between the urban and rural areas, which would be useful for prevention and control of both disease categories in the future.

## 2. Materials and Methods

### 2.1. Data Sources

Official national-level data on annual cause-specific and age-specific mortality rates in urban and rural areas were extracted from Chinese Health Statistical Annual Report (1991–2002), China Health Statistics Yearbook (2003–2012), and China Health and Family Planning Statistics Yearbook (2013–2016), which report the health development and residents’ health status. The mortality data were collected by the Ministry of Health-Vital Registration (MOH-VR) System. The demographic data in urban and rural areas were extracted from China Population Statistics Yearbook (1991–2006), China Population and Employment Statistics Yearbook (2007–2016), which cover age, gender composition of population in different regions (urban and rural). And the population data were obtained by sample survey and census. Data were available for each calendar year from 1990 to 2015 in age groups (<1, 1–4, 5-year age groups to 80–84 and ≥85 years) by region (urban and rural areas). All categorieswere encoded by International Classification of Disease (ICD), Ninth Revision (ICD-9) and Tenth Revision (ICD-10) for CVDs [ICD-9: 390–459, ICD-10: I00–I99], chronic rheumatic heart disease [393–398, I05–I09], hypertensive heart disease [402, I11], ischemic heart disease [410–414, I20–I25], cerebrovascular disease [430–438, I60–I69], RDs [460–519, J00–J99], chronic lower respiratory disease [490–496, J40–J47], pneumonia [480–486, J12–J18], pneumoconiosis [500–505, J60–J65], ICD-9 for data collected prior to 2002 and ICD-10 for data from 2002 onwards.

### 2.2. Statistical Analysis

We calculated age-standardized mortality rates per 100,000 by the direct method for each study year between 1990 and 2015, using the Chinese sixth population census in 2010 as reference [13]. Trends in death rates were analyzed by joinpoint regression. Joinpoint regression is used to compute average annual percent change (APC) and identify joinpoints where significant changes in trends occur. A maximum of three joinpoints was allowed in the analysis. APC was computed using a log-linear model. In addition, 95% confidence intervals were calculated for APC and we used a *p*-value of less than 0.05 as statistically significant. The joinpoint regression was performed by Joinpoint Regression Program, Version 4.5.0.1 (Statistical Methodology and Applications Branch, Surveillance Research Program, National Cancer Institute, Rockville, MD, USA). YLLs quantified the amount of life lost due to premature death from each cause [2]. The basic formula for YLLs is the following for a given cause c, age a, sex s and year t:

YLL(c,s,a,t) = N(c,s,a,t) × L(s,a)

where N(c,s,a,t) is the death number due to the cause c for the given age a and sex s in year t, and L(s,a) is the lost life compared with life expectancy for a death at age a for sex s [14]. The total YLLs for urban and rural areas were obtained by summing the YLL of all age groups. We adopted the recommended standard life expectancy values at birth of 80 years for men and 82.5 years for women. The average age at death was set to the mid-point of each age group, except for the oldest group in whom it was assumed to be 87.5 years [15]. The YLL rate was calculated through dividing the number of YLLs by the number of population in the same calendar year. The method of computing age-standardized YLL rate was similar to that of age-standardized mortality rate. The percent change was defined as the ratio of difference of YLL rates between 1990 and 2015 to the rates in 1990. 

## 3. Results

### 3.1. Trends in Mortality Rates

**Figure 1:**
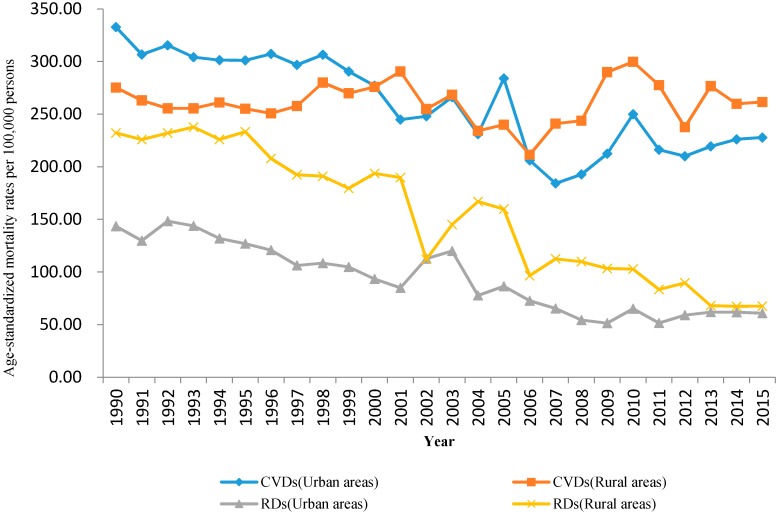
The trend on the age-standardized mortality rates of CVDs and RDs in urban and rural areas during 1990 to 2015.

Age-standardized mortality rates of CVDs and RDs for each year between 1990 and 2015 among urban and rural areas are shown in Figure 1. Continuing decline trends on age-standardized mortality rates were observed for RDs in both urban and rural areas and for CVDs among urban residents. 

However, age-standardized mortality rates of CVDs did not decrease in rural areas from 1990 to 2015. Age-standardized mortality rates from CVDs among rural residents exceeded those among urban residents in 2006. Furthermore, age-standardized mortality rates of RDs for rural residents were higher than those for urban residents during the whole study period, but the rate gap between urban and rural residents has gradually narrowed down in recent years. 

The age-standardized mortality rates of CVDs and RDs at the beginning (1990) and the end (2015) of the study period were shown in Table 1, followed with the average APC during the 26-year period and APC for each sub-period. The joinpoint analysis indicated that, similar to the results in Figure 1, age-standardized mortality rates of CVDs in urban areas declined significantly from 1990 to 2015, with a decrease of 1.9% per year. No change of age-standardized mortality rates of CVDs was observed among rural areas. Based on a further analysis of specific diseases, we noted that age-standardized mortality rates of chronic rheumatic heart disease and cerebrovascular disease decreased in both urban and rural areas during research period. From 1990 to 2015, the age-standardized mortality rates of ischemic heart disease didn’t change in urban areas, but increased significantly in rural areas. However, the age-standardized mortality rate of hypertensive heart disease didn’t change in both urban and rural areas. These might explain the reason of no decline in mortality rates of CVDs among rural areas. 

Age-standardized mortality rates of RDs were trending downward from 1990 to 2015 in both urban and rural areas, with a decrease of 4.2% and 5.3% per year respectively. The steepest decreases were shown from chronic lower respiratory diseases in urban areas (5.6% per year) and from chronic lower respiratory diseases (5.3% per year) and pneumonia (5.6% per year) in rural areas. However, age-standardized mortality rates of pneumonia did not decrease in urban areas, with a significant decline from 1990 to 2005. Additionally, age-standardized mortality rates of pneumoconiosis did not decrease in rural areas.

During the study period, the age-specific mortality rates of CVDs generally decreased in middle and old age people (Table 2), but they increased in the 0- and 1- age group in both urban and rural areas and did not change significantly from 5- to 30- years old groups in urban areas or 5- to 15- and 30- to 40- years old groups in rural areas during research period. Age-specific mortality rates of RDs declined significantly in almost all age groups. The rate of decline from age-specific mortality rates of RDs was greater than those of CVDs.

**Figure 2:**
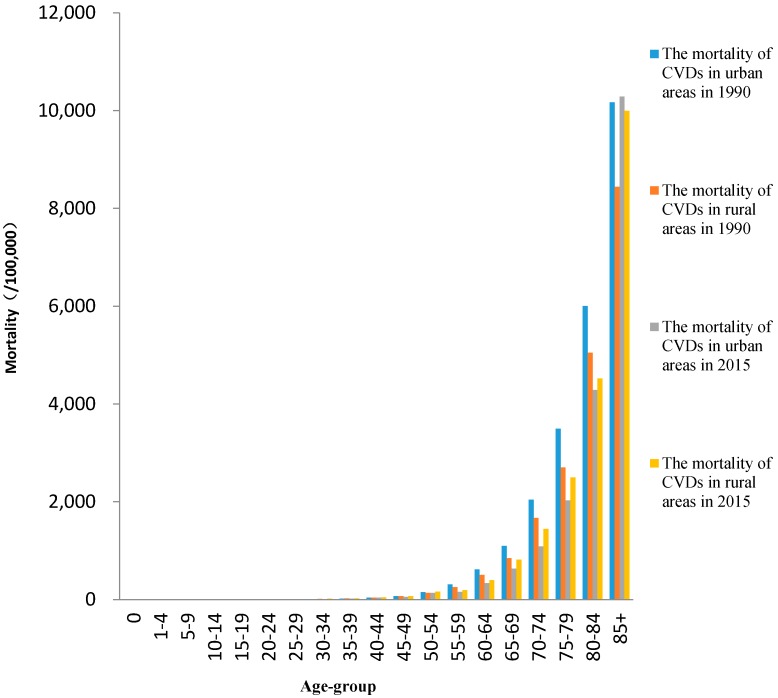
The age-specific mortality rates of CVDs in urban and rural areas in 1990 and 2015.

**Figure 3:**
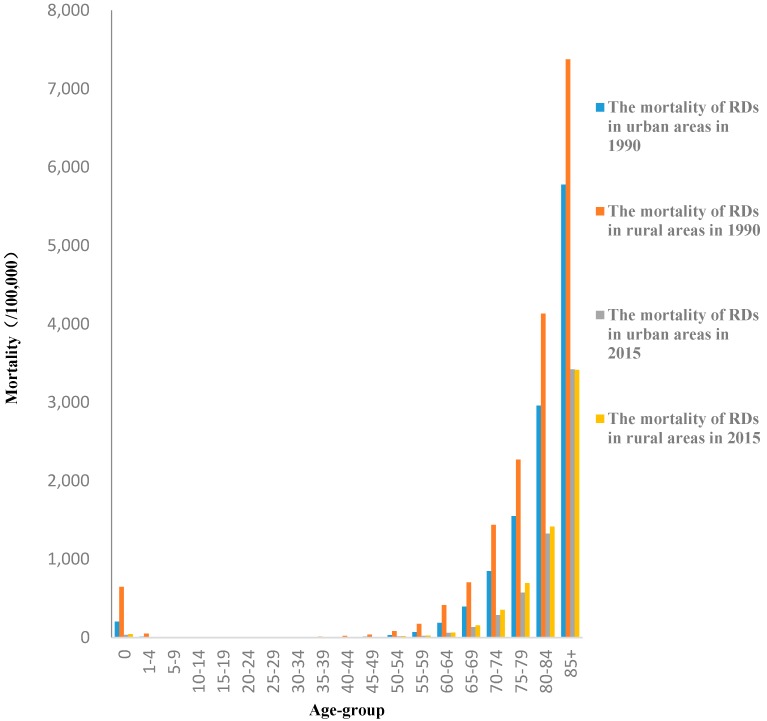
The age-specific mortality rates of RDs in urban and rural areas in 1990 and 2015.

Age-specific mortality rates of CVDs under 45 age group were lower than 100 per 100,000 (Figure 2). They gradually increased with age and were higher than 8000 per 100,000 for those 85 years and older age-group. Age-specific mortality rates of RDs under 45 age group were lower than 50 per 100,000 except for those under 1 age-group, which were 646.6 per 100,000 in rural areas and 203.8 per 100,000 in urban areas in 1990 (Figure 3). The mortality rates gradually increased with age and were higher than 3000 per 100,000 over 85 years old.

### 3.2. YLLs and Age-Standardized YLL Rates

Although the YLLs of CVDs for all population elevated in 2015 when compared with those in 1990, age-standardized YLL rates of CVDs decreased 35.2% in urban areas and 8.3% in rural areas (Table 3). However, it is worth noting that YLLs of chronic rheumatic heart disease have decreased and age-standardized YLL rates of hypertensive heart disease and ischemic heart disease have increased in both urban and rural areas from 1990 to 2015. And elevated percent change of age-standardized YLL rates from ischemic heart disease in rural area (132.9%) was much higher than that in urban area (18.0%). The YLLs of RDs in urban areas increased 43.5% and YLLs in rural areas decreased 78.8% from 1990 to 2015. The age-standardized YLL rates of RDs decreased 64.2% and 79.0% for urban and rural residents respectively. 

## 4. Discussion

In the past 20 years, China has experienced rapid socioeconomic development. During this process, lifestyles, modes of transport and dietary habits have undergone dramatic changes. Meanwhile, medical services and an insurance system have been established and perfected [16]. Influence of those on mortality of CVDs and RDs has been noteworthy. In this study, we found that the trends on age-standardized mortality rates of RDs in both urban and rural areas decreased significantly from 1990 to 2015. Similar trends of CVDs were also observed for urban residents, but not for rural residents. We selected several major diseases of CVDs and RDs for further study. The decline of mortality rates from CVDs was attributed to significantly decreased mortality rates from chronic rheumatic heart disease and cerebrovascular disease. The decline of mortality rates from RDs was attributed to significantly decreased mortality rates from chronic lower respiratory diseases. The total burden of YLLs of CVDs in all residents and of RDs for urban residents increased, conversely total YLLs of RDs for rural residents declined in China from 1990 to 2015. The increased burden might be partly explained by population aging and growth [17,18].

CVDs were the primary cause of death for Chinese population in both urban and rural areas, accounting for about 42% of all deaths in urban areas and 45% of deaths in rural areas in 2015 [19]. Among CVDs, the deaths from chronic rheumatic heart disease, hypertensive heart disease, ischemic heart disease and cerebrovascular disease were 45.4 thousand, 227.6 thousand, 1.5 million, 2.0 million respectively in 2015 [3,20]. In present study, we observed that age-standardized mortality rates from chronic rheumatic heart disease and cerebrovascular disease decreased in both urban and rural areas from 1990 to 2015, which is similar to the results of Global Burden of Disease Study 2013. They found that the age-standardized mortality rates of rheumatic heart disease and cerebrovascular disease decreased 71.2% and 20.9% respectively in China in the past 20 years [21]. Possible reasons for the decline in mortality rate may be better control of risk factors, as well as the improvement of medical service and health system [22].

On the other hand, the age-standardized mortality rates from ischemic heart disease in rural areas, and not in urban areas gradually increased during the same period, which was consistent with previous studies by Jiang and Liu in China [23,24]. However, previous studies observed decline of age-standardized mortality rates from ischemic heart disease in some developed and developing countries over recent decades [25]. Further analyses indicated that the rise in mortality from hypertensive heart disease in urban and rural areas and ischemic heart disease in urban areas started later, after 2007, 2006 and 2004 respectively. Similarly, age-standardized YLL rates of hypertensive heart disease and ischemic heart disease increased in 2015 when compared with those in 1990 in both areas. Many factors such as unhealthy diet, alcohol abuse, hyperlipidemia and high blood pressure are reported to induce hypertensive heart disease or ischemic heart disease in published paper [26,27,28]. China increasingly needs to provide access to high-quality medical services and dietary guidance in communities with increasing rates of CVDs.

Possible reasons for significantly declining death rates from CVDs only in urban areas, and not in rural areas might be attributable to two points. The first is that medical level and service system in urban areas is better than those in rural areas, although both have been improved in the research period [22,29]. The second is that general education level or rate of high education in urban areas is higher compared with rural areas, which might cause rural residents to lack medical knowledge and healthcare consciousness. In addition, rural residents had higher rates of smoking [30], physical inactivity and hypertension compared with urban residents [31,32]. Such a diversity in health between urban and rural areas has been observed worldwide [33,34]. 

As shown in the age-specific mortality rate analysis, the middle and old people were high risk groups in CVDs. Though the mortality rates among middle and old people (over 45 years old) had a significant decrease from 1990 to 2015, the number of death increased because of population aging (3.9 million in 2015, 2.5 million in 1990). Conversely, the mortality rates under 45 years old didn’t show significant decline, but the number of death under 45 years old (99.1 thousand in 2015, 108.4 thousand in 1990) was less than those over 45 years old. It was worth noting that mortality increased under 5 years old. Therefore more efforts are needed in the prevention and control for the CVDs among people of all ages.

In China, RDs were the third leading cause of death, accounting for about 10% of deaths in 2015 [3]. Among RDs, the deaths from chronic lower respiratory diseases, pneumonia and pneumoconiosis were 610.4 thousand, 104.6 thousand and 4.3 thousand respectively in 2015 [3,20]. Further analysis indicated that age-standardized mortality rates from pneumoconiosis in urban areas, pneumonia in rural areas and chronic lower respiratory diseases in both areas declined significantly from 1990 to 2015. Similarly, age-standardized YLL rates of the considered diseases except for pneumoconiosis decreased in rural areas. 

The major risk factors of RDs include tobacco smoke, air pollution, occupational dust exposure and so on [35]. An authoritative report indicated that smoking rates have shown a significant decline, and the smoking-cessation rate increased significantly from 1996 to 2010 [36]. The decreased rate of smoking could contribute to the decline of the mortality from RDs in the whole population. Moreover, the medical service and health care system are constantly improving, which is likely to have a large influence on the decline of mortality from RDs [37]. Though tobacco control efforts have accelerated exceeding expectations in the last few years [38], ambient air pollution in urban area has worsened in recent years [39]. Our results also indicated the age-standardized mortality rates from RDs in urban didn’t change from 2005 to 2015. Similar reports by Zeng and Maji suggested that exposure to particulate matter was significantly connected with elevated risk of dying from RDs [40,41]. The mortality from diseases maybe rise in the near future if the air pollutants cannot be controlled in urban areas. 

To our knowledge, this is the first article to analyze the secular trend on the CVDs and RDs mortality rates in China with the joinpoint regression. We paid more attention to the comparison and analysis of mortality rates trends and YLLs of Chinese urban and rural residents. One limitation of this study is that mortality data were categorized by using ICD-9 codes before 2002 and ICD-10 codes thereafter, which may influence the study's results due to incomplete correspondence. However, the classification of CVDs and RDs in ICD-10 has not change greatly compared with that in ICD-9, and a previous study has shown the comparability of two versions in the analysis of mortality trend [42].

## 5. Conclusions

In total, the age-standardized mortality rates from CVDs and RDs gradually decreased from 1990 to 2015. The decline of the mortality rates from CVDs in urban area was greater than those in rural areas. Conversely, the decline of the mortality rates from RD in rural area was bigger than those in urban areas. With the decline in mortality rates and population growth and aging, increased burden of YLLs from CVDs in both urban and rural residents and from RDs in urban areas were observed. Health promotion and prevention programmes should focus on the major risk factors for CVDs and RDs.

## Figures and Tables

**Table 1:** Joinpoint analysis of age-standardized ^a^ mortality rates from CVDs and RDs in urban and rural areas.

	Mortality Rate (per 100,000)		Total Study Period ^b^		Period 1		Period 2		Period 3		Period 4	
	1990	2015	Average APC (%)	95% CI	Years	APC (%)	Years	APC (%)	Years	APC (%)	Years	APC (%)
CVDs in urban areas	332.7	227.7	−1.9 ^c^	(−2.4, −1.4)	-							
Chronic rheumatic heart diseases	10.4	2.7	−6.1 ^c^	(−6.9, −5.2)	1990–2011	−7.2 ^c^	2011–2015	8.3				
Hypertensive heart disease	8.3	10.8	1.3	(−0.3, 2.9)	1990–2007	−1.6	2007–2015	10.6 ^c^				
Ischemic heart disease	29.1	47.3	0.8	(−0.2, 1.9)	1990–1998	3.3	1998–2006	−5.4 ^c^	2006–2015	8.5 ^c^		
Cerebrovascular disease	181.1	107.2	−2.8 ^c^	(−3.4, −2.2)	1990–2008	−3.7 ^c^	2008–2015	0.8				
CVDs in rural areas	275.2	261.4	0.0	(−0.5, 0.4)	1990–2001	0.7	2001–2006	−4.6	2006–2009	9.19	2009–2015	−1.71
Chronic rheumatic heart diseases	10.8	2.9	−5.9 ^c^	(−6.8, −5.1)	-							
Hypertensive heart disease	9.9	16.5	1.0	(−0.9, 2.9)	1990–2006	−3.2 ^c^	2006–2015	11.2 ^c^				
Ischemic heart disease	18.7	60.0	5.2 ^c^	(3.7, 6.7)	1990–2000	4.1 ^c^	2000–2005	−8.2	2005–2008	37.0	2008–2015	5.7
Cerebrovascular disease	159.5	131.6	−0.6 ^c^	(−1.1, −0.2)	-							
RDs in urban areas	143.5	60.7	−4.2 ^c^	(−4.9, −3.5)	1990–2005	−3.5 ^c^	2005–2008	−14.4	2008–2015	1.9		
Chronic lower respiratory diseases	117.9	44.0	−5.6 ^c^	(−6.7, −4.6)	-							
Pneumonia	13.9	11.7	0.0	(−1.0, 1.0)	1990–2005	−2.7 ^c^	2005–2015	5.0				
Pneumoconiosis	0.8	0.6	−2.3 ^c^	(−3.4, −1.1)	-							
RDs in rural areas	232.1	67.5	−5.3 ^c^	(−6.0, −4.5)	-							
Chronic lower respiratory diseases	199.0	56.3	−5.3 ^c^	(−6.0, −4.5)	-							
Pneumonia	24.2	7.5	−5.6 ^c^	(−6.7, −4.5)	1990–2000	−2.5 ^c^	2000–2003	21.7	2003–2015	−1.4		
Pneumoconiosis	0.5	0.5	0.9	(−1.1, 3.0)	-							

^a^ Standardised to the China sixth population census in 2010. ^b^ Years 1990 to 2015. - No joinpoints identified. Abbreviations: APC, annual percent change; CVDs, cardiovascular diseases; RDs, respiratory diseases. ^c^ Significantly difference from zero (*p* < 0.05).

**Table 2:** APC in age-specific mortality rates from CVDs and RDs in urban and rural areas.

Age Group	CVDs in Urban Areas		CVDs in Rural Areas		RDs in Urban Areas		RDs in Rural Areas	
	Average APC (%)	95% CI	Average APC (%)	95% CI	Average APC (%)	95% CI	Average APC (%)	95% CI
0	3.7 ^a^	(0.4, 7.2)	6.4 ^a^	(2.5, 10.4)	−7.4 ^a^	(−9.1, −5.8)	−13.6 ^a^	(−15.4, −11.5)
1–4	3.8 ^a^	(1.6, 6.0)	3.8 ^a^	(1.1, 6.6)	−3.2 ^a^	(−4.5, −1.8)	−9.1 ^a^	(−11.1, −7.1)
5–9	1.4	(−0.6, 3.4)	1.3	(−0.8, 3.5)	−3.6 ^a^	(−5.0, −2.1)	−10.9 ^a^	(−13.7, −8.0)
10–14	0.6	(−0.9, 2.0)	1.6	(−0.0, 3.3)	−1.9 ^a^	(−3.6, −0.1)	−9.9 ^a^	(−12.8, −6.9)
15–19	2.2	(1.1, 3.3)	0.9	(−0.9, 2.7)	−1.2	(−2.9, 0.5)	−5.8 ^a^	(−9.1, −2.3)
20–24	0.1	(−0.9, 1.2)	−2.0 ^a^	(−3.3, −0.6)	−2.4 ^a^	(−3.9, 0.8)	−7.0 ^a^	(−8.8, −5.1)
25–29	−0.2	(−0.8, 0.5)	−1.8 ^a^	(−3.4, −0.1)	−2.1 ^a^	(−3.0, −1.1)	−8.0 ^a^	(−10.2, −5.7)
30–34	−0.1	(−0.8, 0.6)	−1.1	(−2.7, 0.6)	−3.0 ^a^	(−3.9, −2.0)	−7.4 ^a^	(−9.4, −5.4)
35–39	−0.5 ^a^	(−0.5, −0.0)	−0.0	(−0.7, 0.6)	−3.4 ^a^	(−4.3, −2.5)	−6.7 ^a^	(−7.9, −5.4)
40–44	−0.4	(−0.9, 0.1)	−0.1	(−0.8, 0.7)	−3.4 ^a^	(−4.1, −2.6)	−7.2 ^a^	(−8.5, −5.7)
45–49	−1.3 ^a^	(−2.0, −0.6)	−1.2 ^a^	(−2.4, −0.1)	−3.6 ^a^	(−4.5, −2.7)	−8.1 ^a^	(−9.6, −6.5)
50–54	−1.0 ^a^	(−1.7, −0.4)	−0.8 ^a^	(−1.5, −0.1)	−3.3 ^a^	(−4.3, −2.4)	−7.7 ^a^	(−8.9, −6.6)
55–59	−2.6 ^a^	(−3.2, −2.1)	−0.9 ^a^	(−1.4, −0.5)	−4.6 ^a^	(−5.5, −3.6)	−7.6 ^a^	(−8.8, −6.5)
60–64	−3.4 ^a^	(−4.2, −2.5)	−1.6 ^a^	(−2.1, −1.0)	−5.7 ^a^	(−6.8, −4.6)	−8.1 ^a^	(−9.2, −7.1)
65–69	−3.3 ^a^	(−4.1, −2.5)	−0.9 ^a^	(−1.6, −0.3)	−5.6 ^a^	(−6.6, −4.5)	−7.1 ^a^	(−8.1, −6.0)
70–74	−3.1 ^a^	(−3.8, −2.5)	−1.2 ^a^	(−1.8, −0.6)	−5.5 ^a^	(−6.4, −4.6)	−6.6 ^a^	(−7.5, −5.7)
75–79	−2.3 ^a^	(−2.8, −1.9)	−0.0	(−0.5, 0.5)	−4.7 ^a^	(−5.5, −4.0)	−5.1 ^a^	(−5.9, −4.3)
80–84	−1.5 ^a^	(−2.0, −1.1)	0.4	(−0.1, 0.9)	−4.0 ^a^	(−4.7, −3.3)	−4.3 ^a^	(−5.0, −3.6)
85+	0.0	(−0.6, 0.7)	2.2 ^a^	(1.2, 3.2)	−2.7 ^a^	(−3.3, −2.2)	−2.4 ^a^	(−3.2, −1.5)

^a^ Significantly difference from zero (*p* < 0.05). Abbreviations: APC, annual percent change; CVDs, cardiovascular diseases; RDs, respiratory diseases.

**Table 3:** YLLs and age-standardized YLL rates from CVDs and RDs for urban and rural areas in 1990 and 2015, with percent change between 1990 and 2015.

	YLLs (10,000)			Age-Standardized YLL Rate (per 100,000)		
	1990	2015	Percent Change (%), 1990–2015	1990	2015	Percent Change (%), 1990–2015
CVDs in urban areas	800.9	2385.6	197.9	4365.6	2828.4	−35.2
Chronic rheumatic heart disease	50.9	34.4	−32.4	234.1	40.7	−82.6
Hypertensive heart disease	16.0	95.7	498.1	93.9	113.5	20.9
Ischemic heart disease	166.7	919.0	451.3	920.4	1085.7	18.0
Cerebrovascular disease	428.3	1144.7	167.3	2369.5	1359.1	−42.6
CVDs in rural areas	1983.3	2656.8	34.0	3745.4	3434.3	−8.3
Chronic rheumatic heart disease	152.5	34.4	−77.4	252.3	45.5	−82.0
Hypertensive heart disease	60.3	139.7	131.7	118.8	175.0	47.3
Ischemic heart disease	281.0	941.4	235.0	524.5	1221.4	132.9
Cerebrovascular disease	1069.9	1366.3	27.7	2069.5	1754.6	−15.2
RDs in urban areas	371.0	532.5	43.5	1790.4	640.5	−64.2
Chronic lower respiratory disease	226.8	361.8	59.5	1317.5	432.4	−67.2
Pneumonia	117.6	120.0	2.0	337.1	147.4	−56.3
Pneumoconiosis	2.5	5.6	124.0	12.8	6.8	−46.9
RDs in rural areas	2783.0	590.9	−78.8	3571.0	751.5	−79.0
Chronic lower respiratory disease	1299.8	457.9	−64.8	2511.4	568.1	−77.4
Pneumonia	1420.1	88.1	−93.8	937.7	121.5	−87.0
Pneumoconiosis	5.3	7.7	45.3	10.1	10.5	4.0

Abbreviations: YLL, years of life lost; CVDs, cardiovascular diseases; RDs, respiratory diseases.
